# Day-by-Day Blood Pressure Variability Is Associated With Neurological Functional Outcome After Acute Ischemic Stroke

**DOI:** 10.3389/fneur.2020.566825

**Published:** 2020-11-13

**Authors:** Changqiang Yang, Kai Liu, Yue Song, Shenzhen Gong, Runyu Ye, Zhipeng Zhang, Xiaoping Chen

**Affiliations:** ^1^West China Hospital, Sichuan University, Chengdu, China; ^2^West China Second University Hospital, Sichuan University, Chengdu, China

**Keywords:** acute ischemic stroke, blood pressure variability, neurological functional outcome, cerebral autoregulation, risk factors

## Abstract

**Background:** Increased blood pressure variability (BPV) might be a detrimental factor after acute ischemic stroke. Previous studies on the association between blood pressure variability in the acute ischemic stroke and functional outcome have yielded inconsistent results. We aimed to investigate the impact of day-by-day blood pressure variability within 7 days of onset on functional outcome at 3 months after acute ischemic stroke.

**Methods:** Total 367 patients hospitalized for ischemic stroke within 48 h of onset were enrolled. The acute stage of ischemic stroke was defined as the time period from symptom onset to 7 days. During this period, blood pressure was measured twice daily (respectively, in the morning during 8:00 a.m.−10:00 a.m., in the afternoon between 15:00 p.m. and 17:00 p.m.). Day-by-day blood pressure variability, including standard deviation (SD) and coefficient variation (CV) were derived and compared to functional outcome. We dichotomized function outcome according to mRS score and unfavorable outcome was defined as mRS ≥3.

**Results:** The patients with unfavorable outcome had significantly higher systolic BPV (within 7 days of onset) than those with favorable outcome (15.41 ± 4.59 vs. 13.42 ± 3.95 mmHg for SD, *P* < 0.001; 11.54 ± 3.23 vs. 10.41 ± 2.82 for CV, *P* = 0.001). Multivariable logistic regression analysis revealed that systolic BPV was significantly and independently associated with the 3-month functional outcome [odds ratio (OR) = 1.15, 95% confidence interval (CI): 1.07–1.22, *P* < 0.001 for SD; OR = 1.15, 95% CI: 1.06–1.26, *P* = 0.001 for CV]. In addition, After adjustment for multiple confounding factors, including age, gender, risk factors, stroke features, baseline severity, recanalized therapy, hemorrhagic transformation, pulmonary infection, white blood cell, estimated Glomerular Filtration Rate and mean BP, day-by-day BP variability was significantly correlated with an unfavorable outcome in the top vs. bottom quartile of systolic BPV (OR = 3.33, 95% CI: 1.41–7.85, *P* = 0.006 for SD; OR = 2.27, 95% CI: 1.04–4.94, *P* = 0.037 for CV) during 3-month follow-up. Similar trends were also observed for diastolic BPV. More importantly, incorporating SD of systolic BP into the conventional prediction model could significantly increase the AUC for prediction of 3-month unfavorable outcome after acute ischemic stroke (0.84 vs. 0.86; *P* = 0.0416).

**Conclusions:** Increased day-by-day blood pressure variability of systolic or diastolic BP in the acute ischemic stroke was associated with higher risk for unfavorable outcome at 3 months independent of blood pressure levels. Combining SD of systolic BP with conventional risk factors could improve the prediction of unfavorable outcome.

## Introduction

Hypertension is the most prevalent modifiable risk factor for ischemic stroke and blood pressure reduction is an important goal for stroke risk reduction (1). Optimal blood pressure levels have been well-established for the primary and secondary prevention of ischemic stroke occurrence and recurrence (2). However, the optimal management of blood pressure during the acute stage of ischemic stroke has not been confirmed and remains controversial (3). A series of randomized clinical trials of blood pressure lowering in acute ischemic stroke have been conducted and showed a neutral effect of BP reduction on clinical outcomes (4–8). Recent studies have suggested that the risk of cardiovascular complications may not only depend on the magnitude of blood pressure elevation but also on the presence of increased blood pressure variability, even to a larger extent than average BP values in populations at high cardiovascular risk (9). Therefore, blood pressure variability may be an important predictor of ischemic stroke risk and outcome.

Available evidence about the effect of BPV on outcome after acute ischemic stroke is scarce. Most studies focus on the association between short-term blood pressure variability (BPV) within first 24–72 h after ischemic stroke onset and functional outcome (10–15). Some report that short-term BPV is associated with unfavorable outcome at 3 months after acute ischemic stroke (12–14), whereas others do not find any association (10, 11, 15). A recent systemic review and meta-analysis suggests that this short-term blood pressure variability assessed in the acute stage of ischemic stroke is associated with poor long-term functional outcome (16). However, there are limited data regarding the relationship between mid-term blood pressure variability (day-by-day BPV) and outcome in acute ischemic stroke. The aim of the present study is to investigate whether day-by-day blood pressure variability within 7 days of onset is associated with 3-month functional outcome of patients with acute ischemic stroke.

## Materials and Methods

### Patients

A single-center, retrospective, observational study was performed. The study was approved by the ethic committee of the West China Hospital, Sichuan University, with a waiver of informed consent because of the retrospective nature of this study and minimal risk to participants. We enrolled 367 patients with a first-ever ischemic stroke admitted to the neurological department of West China Hospital, Sichuan University between May 1, 2018 and October 1, 2019, who were admitted to the hospital within 48 h of onset, were hospitalized for >5 days and were functionally independent before stroke attack. The management of patients with AIS was based on the Chinese guidelines for diagnosis and treatment of AIS (17). The exclusion criteria were as follows: (1) the modified Rankin Scale (mRS) score >1 before stroke onset;(2) severe mental disorders, dementia, and seizure; (3) severe systemic diseases and expectancy life span <90 days, such as end-stage heart failure, malignant tumors; (4) history of ischemic or hemorrhagic stroke; (5) estimated glomerular filtration rate <30 ml/min/1.73 m^2^ or acute renal failure; (6) alanine transaminase or aspartate aminotransferase >2.0 upper limit of normal or severe liver disease; (7) incomplete measurement of blood pressure; abnormal SBP or DBP value, where abnormal SBP was defined as either >260 or <70 mmHg; abnormal DBP was defined as either >150 or <40 mmHg. We also excluded patients who died during hospitalization or were discharged just before death as patients' families wanted them to die at home according to the Chinese tradition.

### Data Collection

At admission, demographic features (age and gender), risk factors (hypertension, diabetes mellitus, atrial fibrillation, smoking, and excessive alcohol consumption), history of concomitant cardiovascular disease (coronary heart disease and heart failure) were recorded. Anthropometric parameters (weight and height) were also measured. The severity of stroke was assessed at admission with the National Institutes of Health Stroke Scale (NIHSS) (18). Routine laboratory investigations were tested at admission including hemoglobin, platelet, white blood cell, albumin, estimated Glomerular Filtration Rate, uric acid, triglyceride, total cholesterol, and high density lipoprotein cholesterol. All patients underwent head computed tomography or magnetic resonance imaging at admission and a second brain computed tomography or chest computed tomography was performed if clinically indicated. Ischemic stroke was further classified as large-artery atherosclerosis, cardio-embolism, small-artery occlusion, and unclassified (other determined or undetermined etiology) subtypes (19).

### Blood Pressure Measurement and BP Variability

The acute phase of ischemic stroke was defined as the time period within 7 days of symptom onset (20). SBP and DBP were measured twice daily for 7 consecutive days (respectively, in the morning during 8:00 a.m.−10:00 a.m., in the afternoon between 15:00 p.m. and 17:00 p.m.) and measured three times at each time point and then the average is calculated. Blood pressure was measured in the non-paretic arm, in the supine position, with an automated electronic sphygmomanometer or non-invasive BP monitoring system and entered manually into the electronic medical record as part of the clinical routine. BP values with ≥10 measurements were used to calculate the blood pressure variability indices. Day-by-day blood pressure variability during the acute phase of ischemic stroke was assessed with the SD and coefficient of variation (defined as 100xSD/mean) of SBP and DBP measurements within 7 days of stroke onset (21).

### Outcome Definition

Functional outcome was evaluated by modified Rankin Scale at 3 months after stroke onset (22). Information of functional status was collected via direct or telephone interviews with patients or family members by trained neurologists. We dichotomized function outcome according to mRS score. Unfavorable outcome was defined as mRS score ≥3 and favorable outcome was defined as mRS score ≤2 (15).

### Statistical Analysis

Continuous variables were presented as mean ± SD or median (interquartile range). Categorical variables were expressed as percentage. The baseline characteristics between the two groups were compared using Student's *t*-tests, Mann–Whitney *U*-tests or λ^2^-tests, depending on the appropriate value properties. Binary logistic regression analysis was performed to assess the independent association of each BP parameter with the unfavorable outcome. Variables for adjustments were identified from univariate analyses when their probability values were 0.1 and risk factors confirmed by previous studies. To further evaluate the association between day-by-day BP variability and functional outcome, patients were divided into quintiles (Q1–Q4) according to each blood pressure variability indices. Q1 was used as the reference. Some variables were selected to be included in the logistic regression models. Model 1 was adjusted for age and gender; Model 2 was adjusted for age and gender plus smoking, alcohol consumption, hypertension, diabetes mellitus, atrial fibrillation, coronary artery disease/heart failure, WBC, eGFR, TG, TC, HDL, NIHSS score, TOAST classification, recanalized therapy, hemorrhagic transformation, pulmonary infection, and antihypertensive treatment; In Model 3, mean blood pressure values were included on the basis of Model 2. The dose-response relationships between functional outcome and the BP quintiles were examined by likelihood ratio tests for linear trend. The results of the unfavorable outcome at 3 months were present as odds ratios (ORs) and 95% confidence intervals (CIs). The AUC of the receiver operator characteristic (ROC) curve was computed using the predicted probability of unfavorable outcome. *P* < 0.05 was considered statistically significant. All statistical analyses were conducted using SPSS (IBM version 21.0).

## Results

### Comparison of the Baseline Characteristics

Among the 405 patients who initially met the inclusion criteria, 18 patients were died during hospitalization and 20 patients were lost to follow-up or refused to respond during the follow-up. Thus, 367 eligible patients were enrolled in this study. Overall, the mean age of the study population was 66.6 years and 34.3% were females. The median NIHSS at admission was 7 (interquartile range, 3–14) and 33.8% received recanalized therapy including intravenous thrombolysis or endovascular mechanical thrombectomy. According to the mRS score, 166 patients (45.2%) were defined as unfavorable outcomes at 3 months after ischemic stroke. A comparison of the baseline characteristics was summarized in Table 1. The patients with unfavorable outcomes were more likely to be older, had a higher proportion of atrial fibrillation, suffered more severe neurological deficits at admission, had a higher incidence of pulmonary infection, hemorrhagic transformation and had higher white blood cell counts and lower eGFR.

**Table 1:** Comparison of baseline characteristics by functional status at 3 months.

**Variable**	**Total *n* = 367**	**Favorable outcome *n* = 201**	**Unfavorable outcome *n* = 166**	***P*-value**
Age (years)	66.6 ± 13.5	64.2 ± 14.1	69.5 ± 12.1	<0.001
female (*n*, %)	126 (34.3)	61 (30.3)	65 (39.2)	0.077
BMI (kg/m^2^)	23.4 ± 3.2	23.6 ± 3.3	23.3 ± 3.2	0.325
Smoking (*n*, %)	120 (32.7)	73 (36.3)	47 (28.3)	0.104
Alcohol consumption (*n*, %)	69 (18.8)	41 (20.4)	28 (16.9)	0.389
Hypertension (*n*, %)	211 (57.5)	107 (53.2)	104 (62.7)	0.069
AF (*n*, %)	113 (30.8)	41 (20.4)	72 (43.4)	<0.001
DM (*n*, %)	99 (27)	47 (23.4)	52 (31.3)	0.088
CHD/HF (*n*, %)	95 (25.9)	47 (23.4)	48 (28.9)	0.228
Hemoglobin (g/l)	138.12 ± 19.33	138.80 ± 19.06	137.30 ± 19.67	0.462
Platelet (10^9^/L)	175.37 ± 63.43	179.73 ± 58.23	170.08 ± 69.02	0.147
White blood cell (10^9^/L)	7.91 ± 2.86	7.46 ± 2.58	8.45 ± 3.08	0.001
Albumin (g/l)	41.94 ± 4.11	42.24 ± 4.08	41.57 ± 4.13	0.119
eGFR (ml/min/1.73 m^2^)	84.47 ± 18.47	86.40 ± 18.69	82.13 ± 17.99	0.027
Uric acid (μmol/L)	361.34 ± 103.17	367.67 ± 108.67	353.68 ± 95.86	0.197
TG (mmol/L)	1.68 ± 1.52	1.78 ± 1.63	1.56 ± 1.36	0.181
TC (mmol/L)	4.39 ± 1.04	4.44 ± 1.12	4.33 ± 0.93	0.314
HDL-C (mmol/L)	1.24 ± 0.34	1.22 ± 0.35	1.26 ± 0.34	0.368
NIHSS score	7 (3–14)	4 (2–9)	13 (6–17)	<0.001
TOAST classification				
LAA (*n*, %)	133 (36.2)	61 (30.3)	72 (43.4)	
CE (*n*, %)	104 (28.3)	45 (22.4)	59 (35.5)	
SAO (*n*, %)	96 (26.2)	70 (34.8)	20 (15.7)	
Unclassified (*n*, %)	34 (9.3)	25 (12.4)	9 (5.4)	
Recanalized therapy (*n*, %)	124 (33.8)	67 (33.3)	57 (34.3)	0.84
HT (*n*, %)	60 (16.3)	20 (10)	40 (24.1)	<0.001
Pulmonary infection (*n*, %)	123 (33.5)	37 (18.5)	86 (51.8)	<0.001
Antihypertensive treatment	147 (40.0)	76 (37.8)	71 (42.7)	0.334

*BMI, body mass index; AF, atrial fibrillation; DM, diabetes mellitus; CHD, coronary heart disease; HF, heart failure; eGFR, estimated Glomerular Filtration Rate; TG, triglyceride; TC, total cholesterol; HDL-C„ high density lipoprotein cholesterol; NIHSS, National Institutes of Health Stroke Scale; TOAST, Trial of Org 10 172 in acute stroke treatment; LAA, Large artery atherosclerosis; CE, cardio-embolism; SAO, Small artery occlusion; HT, hemorrhagic transformation*.

### Exploration of Independent Risk Factors for 3-Month Unfavorable Outcome

To explore the independent risk factors for 3-month unfavorable outcome, we selected variables *P* < 0.1 in univariate analysis and risk factors confirmed by previous studies including smoking, alcohol consumption, coronary heart disease, or heart failure, TG, TC, HDL, recanalized therapy, and antihypertensive treatment to perform multivariable logistic regression analysis. It revealed that diabetes mellitus, hypertension, atrial fibrillation, NIHSS score, pulmonary infection, and no recanalized therapy were an independent predictor of unfavorable outcome at 3 months after acute ischemic stroke (Table 2). No recanalized therapy was the best predictor of unfavorable outcome at follow-up (OR = 6.27, 95% CI 3.01–13.09, *P* < 0.001). No association was identified between other variables and unfavorable outcome.

**Table 2:** Multivariable analysis of the unfavorable outcomes.

**Variable**	OR^*^	**95% CI**	***P*-value**
DM	2.14	1.20–3.81	0.009
Hypertension	2.13	1.24–3.65	0.006
AF	2.02	1.12–3.65	0.02
NIHSS score	1.22	1.15–1.29	<0.001
No recanalized therapy	6.27	3.01–13.09	<0.001
Pulmonary infection	2.74	1.49–5.01	0.001

* *variables enrolled in the logistical regression model were age, gender, smoking, alcohol consumption, hypertension, atrial fibrillation, diabetes mellitus, coronary heart disease, or heart failure, eGFR, WBC, TG, TC, HDL, NIHSS score, TOSAT classification, recanalized therapy, hemorrhagic transformation, pulmonary infection, and antihypertensive treatment*.

### Comparison of the BP and BP Variability Indices

Significant differences between patients who were favorable outcome at 3 months and those who were unfavorable were shown in Table 3. The patients with unfavorable outcome had significantly higher day-to-day SBP variability parameters (SBP-SD and SBP-CV) than the patients with favorable outcome. The same finding was true for the DBP variability parameters (DBP-SD and DBP-CV).

**Table 3:** Comparison of SBP and DBP profiles between patients with favorable and unfavorable outcome.

**Variable**	**Favorable outcome *n* = 201**	**Unfavorable outcome *n* = 166**	***P*-value**
SBP-mean (mmHg)	128.77 ± 13.14	133.42 ± 12.93	0.001
DBP-mean (mmHg)	76.36 ± 8.82	76.56 ± 9.00	0.829
SBP-SD (mmHg)	13.42 ± 3.95	15.41 ± 4.59	<0.001
DBP-SD (mmHg)	9.41 ± 2.64	10.36 ± 2.96	0.001
SBP-CV (%)	10.41 ± 2.82	11.54 ± 3.23	<0.001
DBP-CV (%)	12.36 ± 3.28	13.55 ± 3.62	0.001

*CV, coefficient of variation; SD, standard deviation; SBP, systolic blood pressure; DBP, diastolic blood pressure*.

### Continuous BP Variability and 3-Month Neurological Functional Outcomes

In multivariable logistic regression analysis, each BP variability parameters were added to logistic models separately. It demonstrated that SBP-mean, SBP-SD, DBP-SD, SBP-CV, DBP-CV were independently associated with risk of unfavorable outcome at 3 months after acute ischemic stroke, while DBP-mean was no correlation presented in Table 4.

**Table 4:** Multivariable logistic regression analysis of the unfavorable outcomes at 3 months.

**Variable**	**Multivariable logistic regression analysis**		
	**OR**	**95% CI**	***P*-value**
SBP-mean	1.04	(1.02–1.06)	<0.001
SBP-SD	1.15	(1.08–1.23)	<0.001
SBP-CV	1.15	(1.05–1.25)	0.001
DBP-mean	1.01	(0.98–1.04)	0.42
DBP-SD	1.15	(1.05–1.26)	0.03
DBP-CV	1.08	(1.003–1.16)	0.042

*Multivariable logistic regression analysis model included age, gender, smoking, alcohol consumption, hypertension, AF, DM, CAD/HF, eGFR, WBC, TG, TC, HDL, NIHSS score, TOAST classification, Recanalized therapy, HT, Pulmonary infection and antihypertensive treatment*.

### Categorized BP Variability and 3-Month Neurological Functional Outcomes

Patients were divided into quartile groups according to each parameter of day-by-day systolic BP variability. The relationship between systolic BP variability parameters and the risk of unfavorable outcome was further explored in different logistic regression models. In model 1 adjusted for age and gender, SD of SBP in the 4th quintile were positively association with unfavorable outcome (OR = 2.57, 95% CI: 1.37–4.83, *P* = 0.003), compared with that in the 1st quintile (Table 5). In model 2, the association between SD and CV of SBP and unfavorable outcome remain significant (Table 5). After further adjusting for the mean SBP in model 3, compared with those in the 1st quintile, both SD and CV of SBP in the 4th quintile were significantly associated with unfavorable functional outcome (OR = 3.33, 95% CI: 1.41–7.86, *P* = 0.006 for SD; OR = 2.30, 95% CI: 1.06–5.02, *P* = 0.035 for CV) (Table 5). Similar trends were observed in SD and CV of DBP (Table 6).

**Table 5:** Odds ratios for unfavorable outcome according to the quartiles of mean and variability of SBP.

**Variable**	**Model 1**			**Model 2**			**Model 3**		
	**OR**	**95% CI**	***P***	**OR**	**95% CI**	***P***	**OR**	**95% CI**	***P***
SBP-mean Q1 (≤121.46) Q2 (121.47–131.08) Q3 (131.09–140.77) Q4 (≥140.78) *P* for trend SBP-SD Q1 (≤11.23) Q2 (11.24–13.63) Q3 (13.64–17.25) Q4 (≥17.26) *P* for trend SBP-CV Q1 (≤8.63) Q2 (8.64–10.53) Q3 (10.54–12.79) Q4 (≥12.80) *P* for trend	1.00 0.99 1.61 1.97 1.00 1.23 1.58 2.57 1.00 0.89 1.40 1.78	Reference 0.53–1.85 0.87–2.99 1.07–3.63 Reference 0.67–2.88 0.85–2.92 1.37–4.83 Reference 0.48–1.64 0.77–2.56 0.96–3.27	– 0.99 0.12 0.02 0.01 – 0.49 0.14 0.003 0.002 – 0.71 0.26 0.06 0.025	1.00 1.67 2.11 2.98 1.00 1.78 2.57 3.94 1.00 1.14 2.15 2.34	Reference 0.70–3.97 0.85–5.21 1.12–7.89 Reference 0.80–3.95 1.17–5.63 1.71–9.06 Reference 0.52–2.50 1.01–4.57 1.09–5.04	– 0.246 0.104 0.028 0.027 – 0.155 0.018 0.001 0.001 – 0.732 0.045 0.029 0.013	1.00 1.67 2.36 3.33 1.00 1.07 2.09 2.30	Reference 0.74–3.72 1.07–5.21 1.41–7.86 Reference 0.48–2.36 0.97–4.47 1.06–5.02	– 0.211 0.034 0.006 0.004 – 0.86 0.057 0.035 0.014

*Model 1 was adjusted for age and gender; Model 2 was adjusted for Model 1 plus variables including, smoking, alcohol consumption, hypertension, AF, DM, CAD/HF, eGFR, WBC, TG, TC, HDL, NIHSS score, TOAST classification, Recanalized therapy, HT, Pulmonary infection and antihypertensive treatment; In Model 3, SBP-mean was included on the basis of Model 2*.

**Table 6:** Odds ratios for unfavorable outcome according to the quartiles of mean and variability of DBP.

**Variable**	**Model 1**			**Model 2**			**Model 3**		
	**OR**	**95% CI**	***P***	**OR**	**95% CI**	***P***	**OR**	**95% CI**	***P***
DBP-mean Q1 (≤70.07) Q2 (70.08–75.62) Q3 (75.63–82.46) Q4 (≥82.47) P for trend DBP-SD Q1 (≤7.88) Q2 (7.89–9.56) Q3 (9.57–11.38) Q4 (≥11.39) P for trend DBP-CV Q1 (≤10.46) Q2 (10.47–12.66) Q3 (12.67–15.04) Q4 (≥15.05) P for trend	1.00 0.77 0.90 1.31 1.00 1.00 1.71 2.40 1.00 0.82 1.50 1.80	Reference 0.42–1.40 0.49–1.65 0.70–2.42 Reference 0.54–1.84 0.93–3.13 1.30–4.42 Reference 0.44–1.51 0.82–2.74 0.97–3.34	– 0.40 0.73 0.39 0.315 – 0.99 0.81 0.005 0.001 – 0.53 0.17 0.059 0.017	1.00 0.64 0.98 0.99 1.00 1.11 2.15 2.37 1.00 0.91 1.81 2.26	Reference 0.28–1.42 0.43–2.19 0.44–.2.22 Reference 0.50–2.46 0.98–4.72 1.06–5.28 Reference 0.41–2.07 0.82–4.00 0.99–5.14	– 0.278 0.962 0.980 0.755 – 0.784 0.054 0.035 0.015 – 0.822 0.139 0.051 0.018	1.00 1.11 2.16 2.38 1.00 0.90 1.82 2.27	Reference 0.50–2.46 0.98–4.75 1.03–5.46 Reference 0.41–1.99 0.82–4.03 1.001–5.17	– 0.785 0.055 0.041 0.02 – 0.803 0.137 0.050 0.018

*Model 1 was adjusted for age and gender; Model 2 was adjusted for Model 1 plus variables including, smoking, alcohol consumption, hypertension, AF, DM, CAD/HF, eGFR, WBC, TG, TC, HDL, NIHSS score, TOAST classification, Recanalized therapy, HT, Pulmonary infection and antihypertensive treatment; In Model 3, DBP-mean was included on the basis of Model 2*.

#### ROC Analysis

To explore the predictive ability of blood pressure variability and conventional risk factors in acute ischemic stroke, ROC curve analyses were performed (Table 7). The AUC for SBP-SD was maximum (AUC = 0.623) and the best cut-off points was 14.41 mmHg. Conventional prediction models incorporating variables presented in Table 2 were further analyzed. In Model1, the conventional risk factors of diabetes mellitus, hypertension, atrial fibrillation, NIHSS score, pulmonary infection and no recanalized therapy were included. The AUC for the conventional model was 0.844 (95% CI, 0.802–0.879). In Model2, introducing SBP-SD into the Model1 statistically significantly increased the AUC to 0.860 (95% CI, 0.820–0.894; *P* = 0.0416) (Table 8). However, after adding other BP parameters to the conventional risk factors, the AUC did not demonstrate a significantly increased ability (Table 8).

**Table 7:** Blood pressure parameters for the prediction of unfavorable outcome at 3 months.

**Variable**	**AUC**	**95% CI**	***P*-value**	**Cut-off points**
SBP-mean (mmHg) SBP-SD (mmHg) SBP-CV (mmHg) DBP-mean (mmHg) DBP-SD (%) DBP-CV (%)	0.603 0.623 0.597 0.505 0.599 0.597	0.551–0.654 0.571–0.673 0.545–0.648 0.452–0.557 0.547–0.650 0.545–0.647	0.0005 <0.0001 0.0011 0.876 0.0009 0.0012	130.43 14.41 11.01 80.23 9.99 13.66

**Table 8:** Comparison of each blood pressure parameter combined with conventional risk factors in differentiating unfavorable outcome at 3 months.

**Variable**	**AUC**	**95% CI**	**Change in AUC**	***P*-value**
Model 1 Model1+SBP-mean Model1+SBP-SD Model1+SBP-CV Model1+DBP-SD Model1+DBP-CV	0.844 0.853 0.860 0.854 0.849 0.848	0.802–0.879 0.812–0.887 0.820–0.894 0.813–0.888 0.808–0.884 0.808–0.884	– 0.009 0.016 0.010 0.005 0.004	Reference 0.1806 0.0416 0.1152 0.2795 0.2630

## Discussion

Our study showed that increased day-by-day blood pressure variability of systolic or diastolic BP in the acute ischemic stroke was associated with higher risk for unfavorable outcome at 3 months independent of blood pressure levels. In addition, standard deviation of systolic BP had a greater AUC for predicting unfavorable outcome than other blood pressure variability indices. More importantly, our results suggested that incorporating SD of SBP into the prediction model including conventional risk factors could significantly improve the ability to predict 3-month unfavorable outcome after acute ischemic stroke.

Previous studies mainly focused on the effects of short-term blood pressure variability on the prognosis of acute ischemic stroke. The influence of short-term BP variability in acute ischemic stroke remains controversial (10–13). Yong and Kaste (12) studied 793 patients with acute ischemic hemispheric stroke in the ECASS-II trial. It demonstrated that the successive variation of systolic blood pressure within the first 24 h after acute ischemic stroke was inversely associated with favorable outcome at 3 months. Moreover, a large data analysis from the VISTA Collaboration reported similar results (13). However, Zhang et al. (10) assessed the relationship between the ambulatory BPV within 24 h after admission and the 3-month outcome of AIS patients. A greater variability in systolic blood pressure was not correlated with 3-month outcome in patients with acute ischemic stroke. In addition, Tziomalos et al. (11) also found SBP and DBP variability during the first 2 or 3 days after acute ischemic stroke were not associated with the functional outcome at discharge. There were two reasons for contributing to the inconsistent results. First, short-term BPV was obtained by measuring blood pressure many times in a short period of time (24–72 h). These frequent measurements, especially using 24-h ambulatory blood pressure monitoring, with multiple inflation and deflation of the cuff, affected sleep quality of patients and changed the normal circadian rhythm of BP, which might affect the accuracy of blood pressure variability (23). Second, this short-term BPV could not completely rule out the effect of physiological variation of blood pressure and a higher cut-off value might be needed to reflect the pathological variability (23). Therefore, we measured blood pressure at two relatively fixed points (respectively, in the morning during 8:00 a.m.−10:00 a.m., in the afternoon between 15:00 p.m. and 17:00 p.m.) for 7 consecutive days. It was not only reducing the effect of physiological variability of blood pressure to some extent on function outcome, but also eliminating the influence of frequent measurements on sleep quality of patients and thus truly reflected the pathological variability of blood pressure.

In our study, day-by-day blood pressure variability of unfavorable outcome group was significantly larger than favorable outcome group. Previous studies have supported our main finding. Shi et al. (24) aimed to evaluate the predictive significance of day-to-day blood pressure variability during the first 7 days following acute ischemic stroke for 12-month functional outcomes. BP was recorded daily at 7:00 a.m. for the 7 days post stroke. Day-by-day systolic and diastolic BP variability (SD and CV) during the 7 days of hospitalization were higher in patients with poor functional outcome. After further adjusting for the daily mean SBP, the indices of systolic and diastolic BP variability (SD and CV) were not associated with poor outcomes at 12 months. However, our study indicated that increased day-by-day blood pressure variability of systolic or diastolic BP in the acute ischemic stroke was associated with higher risk for unfavorable outcome at 3 months independent of blood pressure levels. There were two previous studies to confirm and strengthen our findings. The Fukuoka stroke registry (15) demonstrated that day-by-day BP variability in the sub-acute stage is associated with the 3-month functional outcome after acute ischemic stroke. However, there are several differences between our studies. It divided the course after onset into acute (first 3 days) and sub-acute (from 4th to 10th days) phases and focused more on the sub-acute phase. During the sub-acute phase, blood pressure was measured once per day at 10:00 a.m. from 4 to 10 days. In addition, Wang et al. (25) aimed to investigate the relation between mid-term blood pressure (BP) variability (BPV) within 7 days of onset and the prognosis in acute stroke patients and reported the similar results. It is a little difference from our study about BP measurement time. Blood pressure was measured every 4 h from days 1 to 7 (the detailed time points were 1 a.m., 5 a.m., 9 a.m., 1 p.m., 5 p.m., and 9 p.m.) in that study.

Another important finding of our study was that standard deviation of systolic blood pressure in the acute phase of ischemic stroke was superior to the mean of systolic blood pressure and other blood pressure variability indices in differentiating unfavorable functional outcomes after 3 months. Kang et al. (26) studied 2,271 patients hospitalized for ischemic stroke within 48 h of onset and found variability of BP, but not average BP in the sub-acute stage of ischemic stroke, is associated with functional outcome at 3 months after stroke onset. It supported our findings to some extent. Standard deviation of SBP, by itself, showed poor sensitivity for predicting unfavorable outcome. However, adding SD of SBP to the model based on conventional risk factors (including diabetes mellitus, hypertension, atrial fibrillation, NIHSS score, pulmonary infection, and no re-canalized therapy) could statistically significant increased the ability to predict 3-month unfavorable outcome. Therefore, SD of SBP could be regarded as a predictive risk factor for clinical outcome of AIS. In clinical practice, we should not only pay attention to blood pressure levels, but more attention to blood pressure variability.

Limitations of our research included the retrospective nature of the study in a single-center. Selected bias might be existed. But due to broad inclusion criteria in our study, the results could be generalized. In addition, we have no access to potential mediators of poor outcome such as early neurological deterioration and recurrent events. The potential pathophysiological mechanism linking day-by-day blood pressure variability to unfavorable outcome was not well-established and needed to be further explored by well-designed clinical studies.

## Conclusion

Increased day-by-day blood pressure variability of systolic or diastolic BP in the acute ischemic stroke was associated with higher risk for unfavorable outcome at 3 months independent of blood pressure levels. Combining SD of systolic BP with conventional risk factors could improve the prediction of unfavorable outcome after ischemic stroke.

## Data Availability Statement

The original contributions presented in the study are included in the article/supplementary material, further inquiries can be directed to the corresponding author.

## Ethics Statement

The studies involving human participants were reviewed and approved by the ethics committee of West China Hospital, Sichuan University. Written informed consent for participation was not required for this study in accordance with the national legislation and the institutional requirements.

## Author Contributions

CY and KL contributed equally to the study concept and design. CY, SG, RY, and ZZ contributed to the acquisition of data. CY and YS contributed to the analysis and interpretation of data. CY drafted the manuscript. XC provided the final approval of the final version of this manuscript. All authors agreed to be accountable for all aspects of the work in ensuring that questions related to the accuracy or integrity of any part of the work are appropriately investigated and resolved.

## Conflict of Interest

The authors declare that the research was conducted in the absence of any commercial or financial relationships that could be construed as a potential conflict of interest.
